# Non‐Empirical Law for Nanoscale Atom‐by‐Atom Wear

**DOI:** 10.1002/advs.202002827

**Published:** 2020-12-07

**Authors:** Yang Wang, Jingxiang Xu, Yusuke Ootani, Nobuki Ozawa, Koshi Adachi, Momoji Kubo

**Affiliations:** ^1^ Institute for Materials Research Tohoku University 2‐1‐1 Katahira Aoba‐ku Sendai 980‐8577 Japan; ^2^ Department of Mechanical System Engineering Graduate School of Engineering Tohoku University 6‐6‐01 Aoba, Aramaki Aoba‐ku Sendai 980‐8579 Japan; ^3^ College of Engineering Science and Technology Shanghai Ocean University No. 999 Hucheng Ring Road Pudong Shanghai 201306 China

**Keywords:** diamond‐like carbon, interfacial bonds, molecular dynamics, nanoscale wear law, wear

## Abstract

Wear of contact materials results in energy loss and device failure. Conventionally, wear is described by empirical laws such as the Archard's law; however, the fundamental physical and chemical origins of the empirical law have long been elusive, and moreover empirical wear laws do not always hold for nanoscale contact, collaboratively hindering the development of high‐durable tribosystems. Here, a non‐empirical and robustly applicable wear law for nanoscale contact situations is proposed. The proposed wear law successfully unveils why the nanoscale wear behaviors do not obey the description by Archard's law in all cases although still obey it in certain experiments. The robustness and applicability of the proposed wear law is validated by atomistic simulations. This work affords a way to calculate wear at nanoscale contact robustly and theoretically, and will contribute to developing design principles for wear reduction.

## Graphical abstract

A non‐empirical wear law for nanoscale contact situations is proposed and then its robustness and applicability on various contact states are validated by atomistic simulations. This work successfully unveils why nanoscale wear behaviors do not generally obey the description by conventional macroscale empirical laws and then contributes to the development of anti‐wear materials.

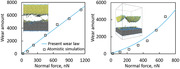

## 1 Introduction

Wear of sliding materials, which results in energy loss and device failures, has long been a crucial area of research.^[^ ^1^, ^2^ ^]^ For conventional macroscale contact, wear is proportional to the applied load (*F_N_*) and sliding distance (*d*) with an empirical wear coefficient (*k*) relating to the material properties and sliding conditions, as described by the famous Archard's law.^[^ ^3^ ^]^ However, Archard's law is an empirical law because of its unclear fundamental physical and chemical origins; hence, a large number of experiments are usually required to obtain the wear coefficient, hindering the development of anti‐wear materials. Furthermore, with the development of nanotechnology in recent years, the nanoscale wear results could not be depicted by Archard's law^[^ ^4^, ^5^, ^6^ ^]^ generally because the establishment of Archard's law is based on macroscopic observations comprising the plastic deformation and wear debris formation which completely differ from the nanoscale behaviors, even though the nanoscale wear behaviors in certain experiments still show Archard‐like behaviors.^[^ ^7^, ^8^ ^]^ Therefore, to benefit the development of nanoscale tribosystems, a non‐empirical wear law that is robustly applicable for various nanoscale contact situations is strongly required.

**Figure 1:**
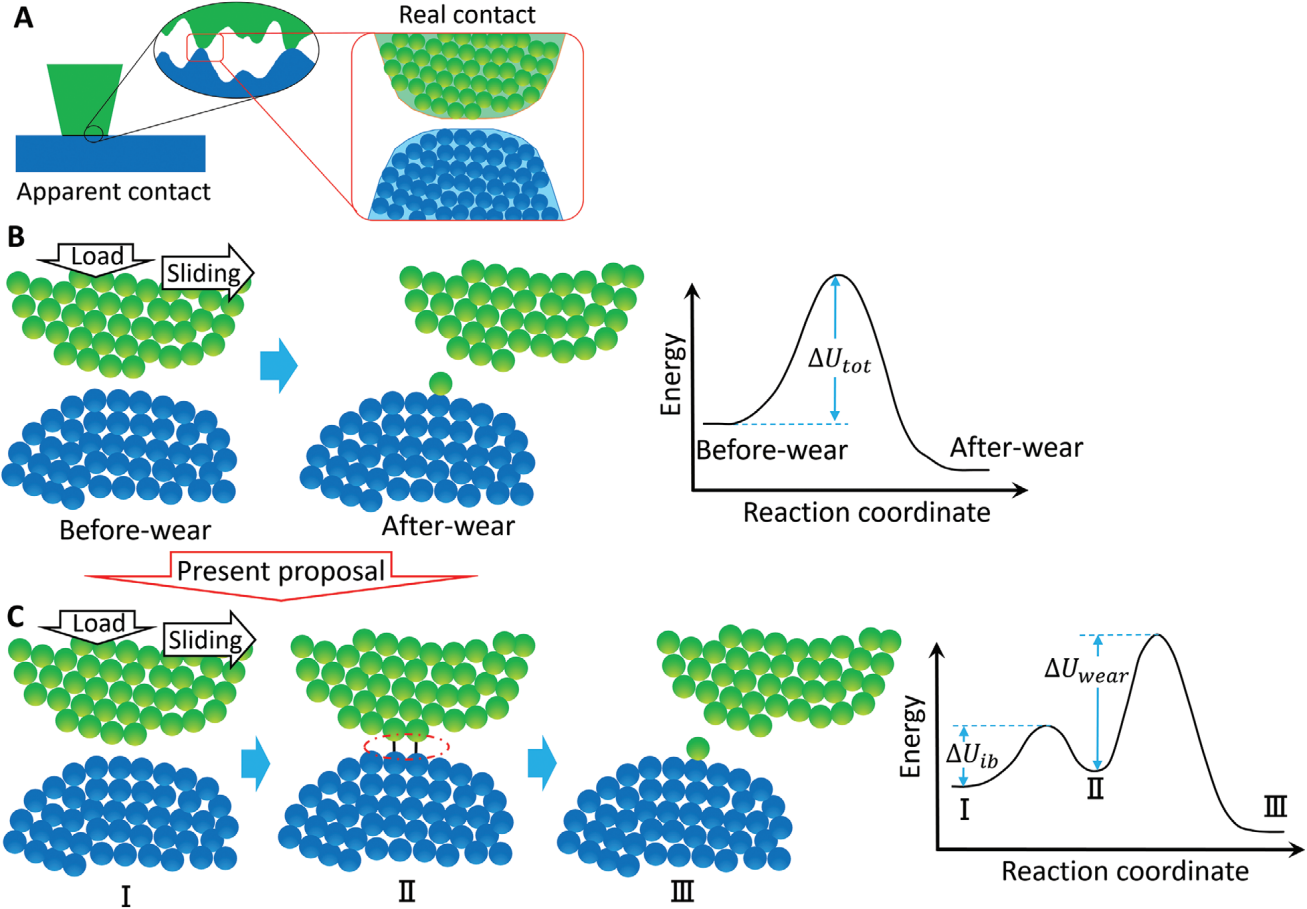
Typical wear process at the atomic scale. A) Typical contact between two substrates. B) Previously proposed wear process as a simple single‐step reaction in which an energy barrier of Δ*U*_tot_ should be overcome from the before‐wear to after‐wear state. C) Presently proposed wear process. Here wear is treated as a two‐step reaction comprising three states: I) before‐wear state, II) interfacial bonding state (atoms on the contact surface reacting with their counterparts and forming the interfacial bonds), and III) after‐wear state (a part of the interfacial bonding atoms are removed from the original surface).

**Figure** **1**A shows a schematic of the typical contact between two substrates. Even though two surfaces are “apparently” in contact from macroscale and microscale view, surfaces are naturally rough with lots of nanoscale asperities and only a small portion of the asperities are “really” in contact.^[^ ^9^ ^]^ It has been widely reported that area of real contact is the main factor affecting tribological behaviors.^[^ ^10^, ^11^, ^12^, ^13^, ^14^ ^]^ At this real contact area, atom‐by‐atom wear process is reported to be the major wear form rather than the abrasive or fatigue wear at macroscale.^[^ ^8^, ^15^, ^16^, ^17^, ^18^, ^19^ ^]^ For the atom‐by‐atom wear process, a wear event should undergo a series of stress‐assisted chemical reactions including bond formation and bond breaking. By assuming the whole wear process is a simple chemical reaction (Figure 1B), the wear rate of contact atom Γ (unit s^−1^) is calculated via reaction rate theory.^[^ ^15^, ^16^ ^]^

(1) $$\begin{array}{c} \Gamma =f_{0}\exp\left(-\frac{\Delta U_{\mathrm{tot}}-W_{\mathrm{tot}}}{k_{B}T}\right) \end{array}$$

Here, *f*_0_ is the attempt frequency,^[^ ^16^ ^]^ which usually ranges from 1.0 × 10^12^ to 1.0 × 10^14^; Δ*U*_tot_ is the total stress‐free activation energy equaling the energy barrier from the before‐wear (corresponding to the real contact state in Figure 1A) to the after‐wear state; *W*_tot_ is the corresponding total external work, which is roughly estimated by multiplying the applied stress component with an effective volume (Δ*V*_act_); and *k_B_* and *T* are the Boltzmann constant and absolute temperature, respectively. By choosing proper values of Δ*U*_tot_ and Δ*V*_act_, this equation reproduced the nanoscale wear results in previous AFM experiments well.^[^ ^5^, ^6^, ^15^, ^16^, ^17^ ^]^ However, these previous works still have several key problems to be tackled: i) there is still controversy about whether normal or shear stress is the dominant component in the external work, *W*_tot_; ii) the fitting parameter Δ*V*_act_ lacks explicit physical and chemical meaning, hindering the establishment of theoretical guidelines for wear reduction; and iii) the robustness of Equation (1) is a big question because it could not explain why the nanoscale wear in certain experiments behaves obeying the description by Archard's law,^[^ ^7^, ^8^ ^]^ whereas does not in others.^[^ ^4^, ^5^, ^6^ ^]^ Here, a non‐empirical and robustly applicable wear law is proposed to address all these problems.

## 2 Results and Discussion

Different to the previous simple treatment of wear, we propose that a wear event should consist of two steps (Figure 1C). In the first step, contact surfaces are compressed together under the load, and hence atoms on the contact surfaces react with their counterparts, forming interfacial bonds that connect the surfaces. We have reported that this interfacial bonding state is an intermediate state in the wear process.^[^ ^19^, ^20^, ^21^ ^]^ In the second step, the atoms with the interfacial bonds are sheared and removed from surfaces during sliding, eventually leading to the wear. These two steps can be regarded as a two‐step stress‐assisted tribochemical reaction, comprising interfacial bond formation (states I and II in Figure 1C) and removal of interfacial bonding atoms (states II and III in Figure 1C), which are assumed to be assisted mainly by the normal stress and shear stress, respectively. Previous works^[^ ^15^, ^16^ ^]^ just simply treat the whole wear process as a single chemical reaction (Figure 1B), and hence the respective roles of normal and shear stress are ambiguous while direct calculations of the corresponding stress‐induced external work are impossible, which significantly hinders us to correctly predict the materials wear. It is crucial to elucidate the respective roles of normal and shear stress and directly obtain the corresponding external works. In this work, we propose that treating the wear process as a two‐step reaction is a more realistic assumption and also is a good choice to reveal the respective roles of the normal and shear stress in the wear, contributing to the theoretical calculation of the corresponding external works. Thus, by applying reaction rate theory to the two‐step reaction, the following non‐empirical wear law is developed (detailed derivations are in the Supporting Information).

(2) $$\begin{array}{c} N_{ib}=N_{rc}\;\exp\left(-\frac{\Delta U_{ib}-W_{\sigma }}{k_{B}T}\right) \end{array}$$

(3) $$\begin{array}{c} N_{\mathrm{wear}}=N_{ib}\;f_{0}\exp\left(-\frac{\Delta U_{\mathrm{wear}}-W_{\tau }}{k_{B}T}\right)t \end{array}$$

*N_rc_* is the number of all atoms in the real contact surface, *N_ib_* is the number of surface atoms that have one or more interfacial chemical bonds, and *N*_wear_ is the number of worn atoms. *N_rc_* can be directly calculated from the real contact area by *N_rc_* =  2*A_rc_*(*F_N_*)/*A*_atom_, where *A*_atom_ is surface area per atom and *A_rc_*(*F_N_*) is the real contact area which is a function of the applied normal load (*F_N_*). Δ*U_ib_* and Δ*U*_wear_ are the activation energies of interfacial bond formation and the removal of interfacial bonding atoms, and *W*_*σ*_ and *W*_*τ*_ are the external work per atom induced by normal stress (*σ*) and shear stress (*τ*), respectively.

In the previous Equation (1), the required external work, *W*_tot_, must be estimated by introducing a fitting parameter, Δ*V*_act_, because of the unclear roles of normal and shear stress; however, because the respective roles of normal and shear stress are cleared presently, we succeed to obtain the corresponding external work, *W*_*σ*_ and *W*_*τ*_ in Equations (2) and (3), respectively, without any extra fitting parameters. Normal stress leads to a compressive deformation of the contact substrates assisting interfacial bond formation; thus, *W*_*σ*_ is assumed as the maximum deformation energy of the real contact surface, that is, $W_{\sigma }=\frac{V_{\mathrm{atom}}}{2E}\;{\left(\frac{F_{N}}{A_{rc}\left(F_{N}\right)}\right)}^{2}$, where *F_N_*/*A_rc_*(*F_N_*) is the normal stress on real contact area, *E* is Young's modulus of the contact material, and *V*_atom_ is average volume per atom. While, *W*_*τ*_ is assumed to be the energy required to stretch an interfacial bond from its equilibrium to the maximum bond length before breaking, which can be directly obtained from the potential energy curve of the bond. Ultimately, expressions of *N_ib_* and *N*_wear_ can be theoretically transformed as follows.

(4) $$\begin{array}{c} N_{ib}=\frac{2}{A_{\mathrm{atom}}}\;\exp\left(-\frac{\Delta U_{ib}}{k_{B}T}\right)\exp\left[\frac{V_{\mathrm{atom}}}{2Ek_{B}T}{\left(\frac{F_{N}}{A_{rc}\left(F_{N}\right)}\right)}^{2}\right]A_{rc}\left(F_{N}\right)\;\; \end{array}$$

(5) $$\begin{array}{ccc} N_{\mathrm{wear}} & = & \frac{2f_{0}t}{A_{\mathrm{atom}}}\;\exp\left(-\frac{\Delta U_{ib}+\Delta U_{\mathrm{wear}}-W_{\tau }}{k_{B}T}\right)\\ & & \times \;\exp\left[\frac{V_{\mathrm{atom}}}{2Ek_{B}T}{\left(\frac{F_{N}}{A_{rc}\left(F_{N}\right)}\right)}^{2}\right]A_{rc}\left(F_{N}\right) \end{array}$$

Here, only Δ*U_ib_* and Δ*U*_wear_ are fitting parameters, which can be extracted by comparing with either experimental or simulation results. Overall, the proposed wear laws successfully make the respective roles of normal and shear stress in the wear process clear and theoretically obtain the corresponding external work with no meaningless parameters, allowing the direct prediction of the materials’ wear. This is a breakthrough because the previous wear law of Equation (1) neither revealed the respective roles of normal and shear stress nor calculated the external work theoretically, even though they are a prerequisite for correctly predicting the wear.

To practically apply Equations (4) and (5) to various nanoscale contact situations, we should first need to know the detailed form of *A_rc_*(*F_N_*), because all other quantities are completely determined by the material type and sliding conditions. In general, *A_rc_*(*F_N_*) is related to the specific contact situation and surface topography. For example, in terms of the contact between two rough surfaces, generally, a linear relation between *A_rc_* and *F_N_* would appear even at nanoscale case.^[^ ^22^, ^23^ ^]^ If we assume that *A_rc_* = *a*_rough_ *F_N_*, normal stress on the real contact area (*F_N_*/*A_rc_*) in Equations (4) and (5) equals to 1/*a*_rough_, which is independent of the load, thereby showing a linear relation of *N*_wear_∝*N_ib_*∝*F_N_*. For *N*_wear_, by substituting *A_rc_* = *a*_rough_ *F_N_* into Equation (5) and writing sliding time (*t*) as the ratio of sliding distance to sliding velocity (*d*/*v*), the expression of *N*_wear_ becomes the following.

(6) $$\begin{array}{ccc} N_{\mathrm{wear}} & = & \frac{2a_{\mathrm{rough}}f_{0}}{A_{\mathrm{atom}}v}\;\exp\left(-\frac{\Delta U_{ib}+\Delta U_{\mathrm{wear}}-W_{\tau }}{k_{B}T}\right)\\ & & \times \;\exp\left(\frac{V_{\mathrm{atom}}}{2E{a_{\mathrm{rough}}}^{2}k_{B}T}\right)F_{N}d \end{array}$$

It shows that *N*_wear_ is proportional to *F_N_d*, which exactly is the similar form as Archard's law ( *N*_wear_ =  *kF_N_d*). Here, it should be noticed that, although *N*_wear_ for this rough‐surface contact shows Archard‐like linear load dependence, the underlying mechanism is completely different from the Archard's law, because the former derives in the interfacial bond formation and atom‐by‐atom wear under light applied loads whereas the latter is established completely based on the macroscale plastic deformation and wear debris formation. Above Equation (6) clearly demonstrates the reason why Archard‐like behaviors emerge in some certain nanoscale experiments. As one another example, for the ball‐on‐disk contact with extremely smooth surfaces, non‐linear relations between *A_rc_* and *F_N_* are reported in experiments.^[^ ^12^, ^13^, ^14^ ^]^ If the non‐linear relation is assumed as *A_rc_* = *a*_ball_ *F_N_* ^*b*^ where exponent *b* depends on the roughness level, the expression of *N*_wear_ is changed to following.

(7) $$\begin{array}{ccc} N_{\mathrm{wear}} & = & \frac{2a_{\mathrm{ball}}f_{0}}{A_{\mathrm{atom}}v}\;\exp\left(-\frac{\Delta U_{ib}+\Delta U_{\mathrm{wear}}-W_{\tau }}{k_{B}T}\right)\\ & & \times \;\exp\left(\frac{V_{\mathrm{atom}}}{2E{a_{\mathrm{ball}}}^{2}k_{B}T}{F_{N}}^{2\left(1-b\right)}\right){F_{N}}^{b}d \end{array}$$

Here, exponent *b* is usually smaller than 1.0, and thus Equation (7) shows a non‐linear load dependence of *N*_wear_, which is differing from Archard‐like behavior because *N*_wear_ does not scale with *F_N_d* anymore. Please notice that, with increasing the roughness, *b* will approach 1.0 gradually and hence the *N*_wear_ − *F_N_* relation is expected to become linear even for nanoscale contact,^[^ ^9^ ^]^ which qualitatively explain why nanoscale wear does not always show Archard‐like behaviors.

**Figure 2:**
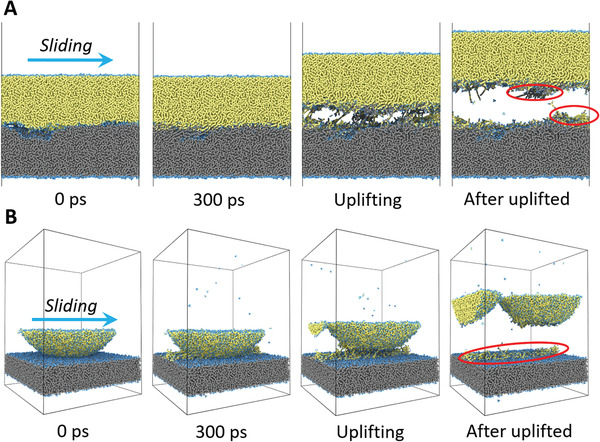
Snapshots of MD simulations. A) Rough‐surface and B) ball‐on‐disk contacts. Blue spheres represent hydrogen atoms, and yellow and gray spheres are carbon atoms in the upper and lower substrates, respectively. The worn atoms are typically indicated by the red circles.

**Figure 3:**
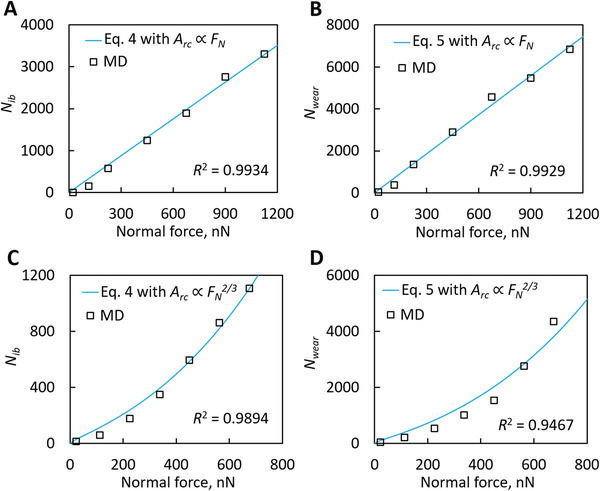
Comparison of our proposed wear law and MD results. Results are for A,B) rough‐surface and C,D) ball‐on‐disk contacts. A,C) Number of interfacial bonding atoms (*N_ib_*) as a function of applied normal force (*F_N_*). B,D) Total wear amount (*N*_wear_) at 300 ps versus *F_N_*. Here *A*_atom_ = 7.104 Å^2^, *V*_atom_ = 6.678 Å^2^, *E* = 280 GPa, *f*_0_ = 10^14^ s^−1^, and *W*_*τ*_ = 0.67 eV are used to give predictions (more details are in the Supporting Information). *R* ^2^ in each panel is the coefficient of determination indicating how well the predictions agree with the MD simulation results.

Next, it is needed to confirm the robustness and applicability of our proposed wear laws for different contact situations such as the aforementioned rough‐surface contact and smooth ball‐on‐disk contact. In details, we should test whether the predicted *N_ib_* and *N*_wear_ well agree with the experiment/simulation results or not under different contact situations. Because the quantities comprising the number of interfacial bonds and *N_ib_* are difficult to be obtained by experiments even using the most advanced experimental instruments, in this work we use a large‐scale reactive molecular dynamics (MD) simulation to calculate *N_ib_* and *N*_wear_ which are then compared with the predictions by Equations (4) and (5). We prepare two simulation setups of a rough‐surface contact and a smooth ball‐on‐disk contact (**Figures** **2**A,B, respectively). For the rough‐surface setup, surfaces have very high self‐affine roughness (relevant details are in the Supporting Information). According to Persson's theory,^[^ ^24^ ^]^ this self‐affine rough‐surface exhibits a linear load dependence of *A_rc_*, even though the simulation model is only tens of nanometers. While the smooth ball‐on‐disk setup is an idealized model of an AFM pin sliding on a disk, and thus a non‐linear load dependence of *A_rc_* is expected. For simulation details, friction simulations of a fully hydrogen‐passivated diamond‐like carbon (DLC)^[^ ^25^, ^26^ ^]^ are performed for 300 ps under various loads. During the friction simulation of both contact models, severe chemical adhesion of surfaces are observed agreeing with the previous works.^[^ ^27^, ^28^ ^]^ When the upper substrate is uplifted after 300 Å of sliding, a large number of surface atoms have transferred to their counterparts, which is exactly the atom‐by‐atom adhesive wear as shown in Figure 1. Furthermore, we also observe the triboemission of gaseous hydrocarbon molecules from friction interface; however, the number of the emitted atoms is even less than 1% of the wear amount of atom‐by‐atom adhesive wear. Besides, we do not observe any cracks and wear debris at friction interface, which are different from Aghababaei's previous simulation results for metal,^[^ ^29^, ^30^ ^]^ because the sizes of surface asperities as well as the surface adhesive forces in present simulations are much smaller than theirs. The above results confirm that for both the rough‐surface and the ball‐on‐disk contact of DLC, atom‐by‐atom adhesive wear is the dominant mechanism rather than crack and debris formation. Then, herein *N_ib_* is obtained as a time average during the last 100 ps (Figure S7, Supporting Information shows how to recognize interfacial bond), and *N*_wear_ is calculated by counting the number of worn atoms after completely lifting the upper substrate. **Figure** **3**A,B shows the simulation results of *N_ib_* and *N*_wear_, respectively, for rough‐surface contact as a function of *F_N_*, while Figure 3C,D are for the ball‐on‐disk contact. For the rough‐surface contact, both *N_ib_* and *N*_wear_ are proportional to *F_N_*, whereas interestingly for the ball‐on‐disk contact *N_ib_* and *N*_wear_ increase with *F_N_* quasi‐exponentially. Additionally, Figure S8, Supporting Information shows the results of *N*_wear_ as a function of sliding distance for the above simulations under two typical applied loads (*F_N_* = 225 and 450 nN). The results show the obvious linearity between *N*_wear_ and sliding distance for both the rough‐surface and ball‐on‐disk contact, which qualitatively agree with the description by Equations (6) and (7).

Then we examine whether our proposed wear law could quantitatively reproduce *N_ib_* and *N*_wear_ for two contact situations, simultaneously. As discussed above, detailed expressions of *N_ib_* and *N*_wear_ are different for the rough‐surface and ball‐on‐disk contact, relying on their *A_rc_* − *F_N_* relations. The present rough‐surface setup gives a linear relation of *A_rc_*∝*F_N_* by Persson's theory^[^ ^24^ ^]^; while for the ball‐on‐disk setup with ideally smooth surfaces, a non‐linear relation of *A_rc_*∝*F_N_* ^2/3^ could be given by the most simple non‐adhesive continuum model (Hertz contact model^[^ ^31^ ^]^), because the presently used DLC surfaces are fully passivated by hydrogen so that the adhesion effect is negligible.^[^ ^32^ ^]^ By putting the relations of *A_rc_*∝*F_N_* (rough‐surface) and *A_rc_*∝*F_N_* ^2/3^ (ball‐on‐disk) into Equations (4) and (5), we can predict *N_ib_* and *N*_wear_ theoretically for both contacts as a function of load. The detailed form of *A_rc_*(*F_N_*) for both rough‐surface and ball‐on‐disk contacts as well as the discussions of all other terms appearing in Equations (4) and (5) is fully shown in the Supporting Information. To ensure that our proposed wear law in Equations (4) and (5) is robustly valid, the predicted *N_ib_* and *N*_wear_ must be able to reproduce the MD results for both rough‐surface and ball‐on‐disk contacts simultaneously, by using the appropriate activation energies (Δ*U_ib_* and Δ*U*_wear_). Practically, Δ*U_ib_* and Δ*U*_wear_ are determined by fitting with the MD results of either rough‐surface or ball‐on‐disk contact, and thus the values extracted from both contacts should be as close as possible because the activation energies only depend on the material type but not on the contact situation. The previous wear laws (including Archard's law and Equation (1)) are unable to reproduce the wear results for both the rough‐surface and ball‐on‐disk contacts simultaneously, indicating that they are not robustly applicable (see Figure S6 and relevant discussions in Supporting Information). However, the presently predicted *N_ib_* and *N*_wear_ by Equations (4) and (5) agree very well with the MD results for both contacts (Figure 3). Meanwhile, the extracted activation energies for the rough‐surface contact are Δ*U_ib_* = 0.032 eV and Δ*U*_wear_ = 0.917 eV, and those for the ball‐on‐disk contact are Δ*U_ib_* = 0.032 eV and Δ*U*_wear_ = 0.905 eV. The extracted activation energies for both contacts show close agreement. Overall, our proposed wear law succeeds to reproduce the wear for both the rough‐surface and smooth ball‐on‐disk contacts simultaneously by using almost the same activation energies, hence proving that our proposed wear law is indeed effective and robust.

## 3 Conclusion

In summary, we proposed a non‐empirical and robustly applicable wear law for the nanoscale contact situations, which makes several breakthroughs in the fundamental study of wear. Our work succeeds to theoretically reveal that the reasons why the nanoscale wear results in certain experiments behaves obeying the description by Archard's law while does not in others, stem from the different load dependences of real contact area. Then, the applicability and robustness of the proposed wear law at nanoscale contacts are successfully proved by the subsequent large‐scale atomistic simulations.

## 4 Simulation Details

The reactive force field (ReaxFF)^[^ ^33^ ^]^ is used to perform MD simulations. ReaxFF parameters used presently are developed in our previous work.^[^ ^27^ ^]^ Since ReaxFF is a kind of bond‐order‐based reactive potential, herein we use the bond order to identify whether two atoms are bonded or not with a cutoff bond order value of 0.5. We use quick‐quenching method to construct DLC bulk models. The ratio of *sp^3^*‐, *sp^2^*‐, and *sp*‐hybridized carbon atoms in the obtained DLC bulk are about 39.4%, 60.4%, and 0.2%, respectively. Then, the DLC bulk is cleaved into the required shapes, and the after‐cleaved DLC is relaxed in a hydrogen gas environment with a pressure of 1000 atm to make the reactive DLC surfaces passivated by hydrogen terminations. After relaxation, the hydrogen molecules are removed.

Figure S1A,B, Supporting Information shows the simulation models of rough‐surface and ball‐on‐disk setup, respectively. For rough‐surface setup, two DLC substrates with self‐affine roughness are packed with each other. The size of simulation box is 150  ×  150  ×  300 Å^3^. For ball‐on‐disk setup, a smooth DLC ball with a radius of 8 nm slides against a smooth DLC disk. The box size is also 150  ×  150  ×  200 Å^3^. Each of DLC model has been relaxed in a high‐pressure hydrogen gas to passivate the surface by hydrogen terminations, as shown in Figure S2, Supporting Information. During sliding simulation, the bottom layer atoms in the lower DLC substrate keep rigid and stationary, while the top layer atoms in the upper DLC also keep rigid but are slid along the *x*‐direction with 100 m s^−1^. To see the load dependence of wear, various normal loads are applied to the upper DLC substrates during the sliding. We keep the layers (with a thickness of 0.5 nm) neighboring to the rigid layers at 300 K by using the Berendsen thermostat.^[^ ^34^ ^]^ Velocity‐Verlet algorithm^[^ ^35^, ^36^ ^]^ is used to calculate the motion of each atom with a time step of 0.25 fs step^−1^. All simulations are performed for totally 300 ps, and then, the upper DLC substrates are lifted up during the sliding with 100 m s^−1^ along *z*‐direction to investigate the wear.

## Conflict of Interest

The authors declare no conflict of interest.

## Author Contributions

M.K. supervised the research. Y.W. designed and performed the simulations, analyzed the data, and wrote the manuscript. J.X. prepared the simulation code framework. All authors participated in the interpretation of the data and discussion of the manuscript.

## Supporting information

Supporting Information

## Data Availability

All data needed to evaluate the conclusions in the paper are present in the paper and/or the Supporting Information. Additional data are available from the authors upon request.
