# Supplementary material for: Non‐Empirical Law for Nanoscale Atom‐by‐Atom Wear
Source: Adv Sci (Weinh). 2020 Dec 7;8(2):2002827. doi: 10.1002/advs.202002827 (PMC7816698; doi:10.1002/advs.202002827)
Supplement: Supplementary file 1 — Supporting Information [file ADVS-8-2002827-s001.pdf]

© 2020 Wiley-VCH GmbH

ADVANCED SCIENCE
Open Access

Supporting Information

for *Adv. Sci.,* DOI: 10.1002/advs.202002827

Non-empirical law for nanoscale atom-by-atom wear

*Yang Wang, Jingxiang Xu, Yusuke Ootani, Nobuki Ozawa, Koshi Adachi, and Momoji Kubo**

Supporting Information for

Non-empirical law for nanoscale atom-by-atom wear

Yang Wang[1, 2], Jingxiang Xu[3], Yusuke Ootani[1], Nobuki Ozawa[1], Koshi Adachi[2], and Momoji Kubo[1, *]

1. Self-affine roughness

The self-affine rough surfaces with the fractal nature are generated based on the following equation[1].

$$z(x,\ y) = L\left(\frac{G}{L}\right)^{D-2}\sqrt{\frac{\ln\gamma}{M}}\sum_{m=1}^{M}\sum_{n=0}^{n_{max}}\gamma^{(D-3)n}\left(\cos\phi_{m,n} - \cos\left[\frac{2\pi\sqrt{x^2+y^2}}{L}\cos\left(\tan^{-1}\left(\frac{y}{x}\right)-\frac{\pi m}{M}\right)+\phi_{m,n}\right]\right) \quad \text{(S1)}$$

$z(x,\ y)$ is the height relating to the planar coordinates of x and y. L is the length scale. G is the height scaling factor which directly relates to the roughness of surface. D is fractal dimension and we have the Hurst exponent of $H = 3 - D$. γ and M are the parameters as described in Reference[1]. n_{max} is given by $n_{max} = \mathrm{int}[\log(L/L_s)\ /\log(\gamma)]$ where L_s is cutoff length approximatelly equaling the lattice parameter of solid. $\phi_{m,n}$ is taken randomly obeying to the normal distribution. However, surfaces created by above equation cannot be directly applied to MD simulations because the periodic boundary condition (PBC) of MD requires $z(0,\ y) = z(L,\ y)$ and $z(x,\ 0) = z(x,\ L)$. To make it applicable for PBC, above equation is modified by using $a(x) = L(k_x x/L)^{(\sin\pi x/L)^2}$ and $b(y) = L(k_y y/L)^{(\sin\pi y/L)^2}$ to replace x and y, respectively, where k_x and k_y are parameters to generate different shapes of $a(x)$ and $a(y)$. For any value of k_x and k_y, there are always $a(0) = a(L)$ and $b(0) = b(L)$, and hence we have $z(0,\ y) = z(L,\ y)$ and $z(x,\ 0) = z(x,\ L)$. Table S1 shows the values of each parameter used to create the rough surfaces for MD simulations.

2. Derivation of Eq. 2 to 7

For a wear event as described in Fig. 1, real contact atoms should react with their counterpart forming the interfacial bonds at the first step, and then those atoms with interfacial bonds are removed from surface, eventually leading to a wear. We firstly should use reaction rate theory to calculate the number of atoms with interfacial bonds (N_{ib}) from the number of all atoms in the real contact area (N_{rc}). Conventionally, reaction rate (r) for any general chemical reaction is given as $r = b\exp\left(-\frac{\Delta U_{act}-W}{k_B T}\right)$, where b is attempt frequency (e.g. for the gas/liquid phase reaction, it is the collision frequency among reactant molecules), ΔU_{act} is stress-free activation energy, and W is external work. For any given real contact atom, it has an opportunity to form interfacial bond with a probability of $\exp\left(-\frac{\Delta U_{ib}-W_{\sigma}}{k_B T}\right)$ for one reaction attempt, where ΔU_{ib} and W_{σ} are the corresponding activation energy and external work, respectively (Fig. 1C), and thus N_{ib} can be calculated as $N_{ib} = \int N_{rc} b\exp\left(-\frac{\Delta U_{ib}-W_{\sigma}}{k_B T}\right)\mathrm{dt}$. N_{rc} can be directly obtained from the real contact area (A_{rc}), that is, $N_{rc} = 2A_{rc}/A_{atom}$ where A_{atom} is average area per atom. Because generally there is little change in the nominal contact area for a given tribopair during the friction, we reasonably assume that both A_{rc} and N_{rc} are independent of time. Furthermore, for the contact between two solids, it is assumed that surface atoms attempt to form interfacial bonds for only once from the non-contact to contact state, that means $\int b\mathrm{dt} = 1$. Thus, expression of N_{ib} can be simplified as $N_{ib} = N_{rc}\exp\left(-\frac{\Delta U_{ib}-W_{\sigma}}{k_B T}\right)$ which corresponds to the Eq. 2.

When N_{ib} is obtained, we can calculate the number of worn atoms (N_{wear}) easily. For the wear problem, the removal of atoms with interfacial bonds occurs continuously during the friction, and thus, N_{wear} is a time cumulative quantity which is given as $N_{wear} = \int N_{ib} b\exp\left(-\frac{\Delta U_{wear}-W_{\tau}}{k_B T}\right)\mathrm{dt}$, where ΔU_{wear} and W_{τ} are the corresponding activation energy and external work, respectively (Fig. 1C). Here, b is written as f_0 to specific the attempt frequency of the removal of interfacial bonding atoms. As reported

previously[2], f_0 is usually as a constant ranging from 1.0×10^{12} to 1.0×10^{14}. Thus, N_{wear} can be rewritten as $N_{wear} = N_{ib} f_0 \exp\left(-\frac{\Delta U_{wear} - W_\tau}{k_B T}\right) t$ which is the Eq. 3.

Because the contact substrates are compressed and deformed under the applied force (F_N), W_σ should equal to the maximum deformation energy of the contact surface monolayer, that is $W_\sigma = \Delta d_\sigma F_N / N_{rc}$ where F_N / N_{rc} is the applied normal force on each real contact atom and Δd_σ denotes the deformation of surface monolayer. Through the elastic deformation analysis on the surface monolayer, we have that $\Delta d_\sigma = F_N t_{atom} / E A_{rc}$, where E is Young's modulus of contact material (here we assume that E for both sides of substrates are the same) and t_{atom} indicates the thickness of surface monolayer which is given by V_{atom} / A_{atom} (V_{atom} is average volume per atom). Then, for the W_τ, we assume W_τ as the required energy of stretching an interfacial bond from its equilibrium to the maximum bond length before breaking, which can be directly obtained from the potential energy curve of the bond. Fig. S3 shows the details of obtaining W_τ where the relevant descriptions and discussions are in the following Section 5 and 6. Thus, Eq. 4 and 5 are deduced by substituting W_σ and W_τ into Eq. 2 and 3.

Last, to obtain the final expressions of N_{ib} and N_{wear} for both rough-surface and ball-on-disk contact, the key is to understand the relation between A_{rc} and F_N because the other terms appearing in the wear law (A_{atom}, V_{atom}, E, W_τ, and f_0) are only relating to specific material type but independent to specific contact situations. Detailed values and discussions of these terms for DLC are available in the following sections. For rough-surface contact with self-affine roughness, the surfaces have been fully passivated by hydrogen terminations and hence we can use the non-adhesive Persson's contact model[3] to estimate A_{rc} with F_N.

$$A_{rc}(\lambda) \approx \frac{4}{q_0 h_0 E^*} \left(\frac{1-H}{\pi H}\right)^{\frac{1}{2}} \left(\frac{\lambda}{L}\right)^{1-H} F_N \qquad \text{(S2)}$$

This equation is valid especially for the elastic and low-adhesion limit according to Johnson's assumption[4]. Where λ is the length scale, $q_0 = 2\pi/\lambda$ is the wave vector, and h_0 is the rms surface roughness of surface. For our generated self-affine rough surfaces, Fig. S4 shows the h_0 as a function of

the length scale λ. $E^* = [(1-{\nu_1}^2)/E_1 + (1-{\nu_2}^2)/E_2]^{-1}$ is the effective elastic modulus of contact pairs where E_1 and E_2 are Young's modulus and ν_1 and ν_2 are Poisson's ratios of each side of substrate. In present simulations, materials for both upper and lower substrates are the same, and thus we have E_1 = E_2 = E, and ν_1 = ν_2 = ν. Then, H is the Hurst exponent, which is 0.5 for the present rough surfaces. λ is length scale, which is equal to the size of simulation box (L) herein because the whole surface is in contact. Thus, for H = 0.5 and λ = L, we have $A_{rc}(L)$ = $4F_N/q_0h_0\sqrt{\pi}\,E^*$, where $4/q_0h_0\sqrt{\pi}E^*$ is exactly the a_{rough} in Eq. 6.

While for the ball-on-disk contact, because the surfaces of both ball and disk are smooth and fully passivated, obviously A_{rc} equals to the apparent contact area, and thus the non-adhesive Hertz contact model[5] could be used to estimate the A_{rc}, that is,

$$A_{rc} = \pi(3R/4E^*)^{2/3}{F_N}^{2/3} \tag{S3}$$

where ball radius R is 8 nm for present model. Thus, by substituting Eq. S3 into the wear law, Eq. 7 is deduced where $\pi(3R/4E^*)^{2/3}$ is exactly the a_{ball}.

3. Determination of A_{atom} and V_{atom}

The average volume per atom, V_{atom}, can be simply calculated from the density of the diamond-like carbon (DLC) bulk, ρ. We have V_{atom} = $M_C/\rho N_A$ where N_A is Avogadro constant and M_C is molar mass of carbon. Then, the average surface area per atom A_{atom} is calculated by using a DLC bulk with a size of $100 \times 100 \times 100$ Å^3. We assume N_{bonds} as the number of bonds across a plane, and thus, A_{atom} can be obtained by A_{atom} = $L^2/\langle N_{bonds}\rangle$, where L = 100 Å is the length of the DLC bulk. As the results, we have V_{atom} = 6.678 Å^3 and A_{atom} = 7.104 Å^2 for presently used DLC samples.

4. Mechanical properties of DLC

To obtain the Young's modulus of DLC, the tensile test is performed by using above DLC bulk with a size of $100 \times 100 \times 100$ Å^3. The DLC bulk is stretched along x-direction with a strain rate of 10^9 s^{-1}

while the y- and z-direction are relaxed during the tensile test. Fig. S5 shows the simulated stress-strain curve, and Young's modulus could be obtained as the slope of the stress-strain relation. Furthermore, Poisson's ratio could be obtained by taking the average ratio of the transverse strain (along y- and z-direction) to axial strain (along x-direction) during tensile simulation. Before the tensile strain of 0.1, DLC bulk does not show obvious yield, and thus, the Young's modulus (E) and Poisson's ratio (ν) are calculated. As the result, we have E = 280 GPa while ν = 0.18.

5. Estimation of W_τ and f_0

As described in manuscript, we assume that W_τ equals to the required energy of stretching an interfacial bond from its equilibrium to the maximum bond length before breaking. For the tested DLC, the interfacial C-C bond breaking process is schematically shown in Fig. S3A. The slide of upper substrate leads to the elongation of the interfacial bond. When the bond is elongated to its maximum length, either of the interfacial bond breaking or wear event will occur. Thus, the problem of estimating W_τ is how to determine the maximum bond length before breaking and the corresponding energy.

Here we perform extra reactive MD simulations to find the maximum C-C bond length during sliding condition. Figs. S3B and S3C show the sliding behaviors of two modified diamond surfaces with totally 8 interfacial bonds at the initial. In Fig. S3B, the diamond surfaces are fully passivated by hydrogen terminations, so that the breaking of interfacial bond will lead to the formation of dangling bonds on the surface; while in Fig. S3C, the hydrogen terminations near the interfacial bonds are removed, and thus, the breaking of interfacial bond will lead to a surface reconstruction generating the carbon-carbon double bonds. These two models are able to represent most conventional surface chemical reactions when interfacial bond breaking occurs. Then, we monitor the bond lengths of all 8 interfacial bonds during the sliding and plot them in Figs. S3D and S3E, corresponding to S3B and S3C, respectively. In both simulations, we observe that the bonds are elongated gradually and then the bong lengths increase suddenly indicating the bond breaking. Insert in Figs. S3D and S3E are the magnification around the

sudden increase of bond length. The two inserts clearly show the critical bond length for bond breakings (maximum bond length), that is roughly in the range of 1.8-1.9 Å. Furthermore, in the radial distribution function (RDF) of DLC as shown in Fig. S3F, the first peak is attributed to the C-C bond and we find the first peak drops to zero after 1.8 Å, indicating that C-C bonds in DLC bulk cannot be elongated higher than 1.8 Å. Overall, it is reasonable to assume 1.8 Å as the maximum bond length.

Next, we need to know the required energy to elongate a C-C bond from its equilibrium to the maximum bond length. Fig. S3G shows the potential curve of C-C bond in an ethane molecule, which is obtained from the first-principle calculations. By taking 1.8 Å as the maximum bond length, we obtained that W_τ = 0.67 eV.

On the other hand, attempt frequency f_0 in principle is related to the atomic vibration of the focused system, and the details can be found in previous literatures[2,6]. Similar to the W_τ, f_0 is not easy to rigorously calculated and thereby it is roughly estimated. In many previous literatures regarding the DLC, f_0 usually takes a value of 10^{13} (see Reference[7]) or 10^{14} (see Reference[8–10]). In this work, the f_0 = 10^{14} is employed.

6. Effect of W_τ and f_0 on the calculation of wear

Here we discuss the effect of W_τ and f_0 on the calculation of wear. It is obviously that W_τ and f_0 are only related to the calculation of N_{wear}, and thus the different W_τ and f_0 will only change the extracted value of ΔU_{wear} after fitting with MD simulation results.

Firstly, the effect of W_τ on the extracted value of ΔU_{wear} is investigated. When the f_0 is kept to 10^{14} while W_τ is varied continuously, we find that for both rough-surface and ball-on-disk contacts the extracted value of ΔU_{wear} increases as the W_τ increases. For example, for the W_τ of 0.06, 0.31, 0.67, and 1.09 eV (corresponding to the maximum C-C bond length d_{max} of 1.6, 1.7, 1.8, and 1.9 Å, respectively),

the correspondingly obtained ΔU_{wear} are 0.3073, 0.5573, 0.9173, and 1.3373 eV for rough-surface contact and 0.2945, 0.5445, 0.9042, and 1.3245 eV for ball-on-disk contact.

Then, in terms of the effect of f_0, when the W_τ is assigned to 0.67 eV while f_0 is varied from10^{12} to 10^{15}, we also observe the increase of extracted ΔU_{wear} with the increasing f_0. In details, for the f_0 of 10^{12}, 10^{13}, 10^{14}, and 10^{15}, the extracted values of ΔU_{wear} are 0.7982, 0.8577, 0.9173, and 0.9769 eV respectively for rough-surface contact, while they are 0.7853, 0.8449, 0.9042, and 0.9641 eV for the ball-on-disk contact.

Although above results show that the value of ΔU_{wear} is strongly relying on the W_τ and f_0, it should be noticed that, to demonstrate the applicability of our proposed wear law, we need to focus on the comparison of extracted ΔU_{wear} between rough-surface and ball-on-disk contact rather than the abslute value of the activation energy. Thus, above results show that, for any W_τ and f_0, the extracted ΔU_{wear} for both contact situations are very close (all the differences are within 1-2%), indicating the very goold applicability of our proposed wear law. In this work, we reasonably but not rigorously estimate that W_τ = 0.67 eV and f_0 = 10^{14}, and the corresponding ΔU_{wear} is about 0.9 eV, showing very good agreement with the existing expriementally obtained activation energies which range from 0.8 to 1.0 eV (see Reference[7,9–11]).

7. Comparison with the previous wear laws

For the Archard's law, it is obvious that Archard's law cannot describe the quasi-exponential load dependence of wear for the ball-on-disk contact because the expression of Archard's law only allows a linear load dependence of wear.

Then, for the previously reported nanoscale wear law of Eq. 1 in manuscript, we compare it with the MD simulation results to test its applicability for each contact situation. In these previous works, the whole wear process is assumed as a one-step reaction, and thus the wear rate of any given contact atom, Γ (unit s^{-1}), can be calculated by the following simple equation.

$$\Gamma = f_0 \exp\left(-\frac{\Delta U_{tot} - \sigma \Delta V_{tot}}{k_B T}\right) \tag{S1}$$

f_0 is the attempt frequency, ΔU_{act} is the stress-free activation energy for the entire wear process, $\sigma \Delta V_{tot}$ is the external work where σ is external normal stress and ΔV_{tot} is effective volume as a fitting parameter. It is able to calculate the number of worn atoms (N_{wear}) from wear rate by multiplying it to the real contact atoms (N_{rc}) and sliding time (t).

$$N_{wear} = N_{rc}\Gamma t = \frac{2A_{rc} f_0 t}{A_{atom}} \exp\left(-\frac{\Delta U_{act} - F_N \Delta V_{act}/A_{rc}}{k_B T}\right) \tag{S2}$$

Here ΔU_{act} and ΔV_{act} are fitting parameters which are needed to be determined by fitting with the MD simulation results. Thus, by taking the expression of A_{rc} as a function of F_N into above equation, the relations between N_{wear} and F_N for both the rough-surface and ball-on-disk contact can be predicted. Fig. S6A and S6B in manuscript show the comparison results of N_{wear} for rough-surface and ball-on-disk contact, respectively. Black open squares are the MD results and red open triangles are the predicted N_{wear} based on Eq. S2. For the rough-surface contact, the extracted values are ΔU_{act} = 0.2965 eV and ΔV_{act} = 1.0001 Å^3. Meanwhile for the ball-on-disk contact, the extracted values are ΔU_{act} = 0.3581 eV and ΔV_{act} = 0.9233 Å^3 which are quite different from rough-surface contact. Furthermore, for the rough-surface contact, if we plot the prediction results using ΔU_{act} = 0.3581 eV and ΔV_{act} = 0.9233 Å^3 which are same with the ball-on-disk contact (orange open diamond in Fig. S6A), we observe that the obtained results are much lower than the MD results, showing a huge difference from the MD simulation results. All above results clearly indicate that this previous wear law cannot be applied to both rough-surface and ball-on-disk contacts simultaneously.

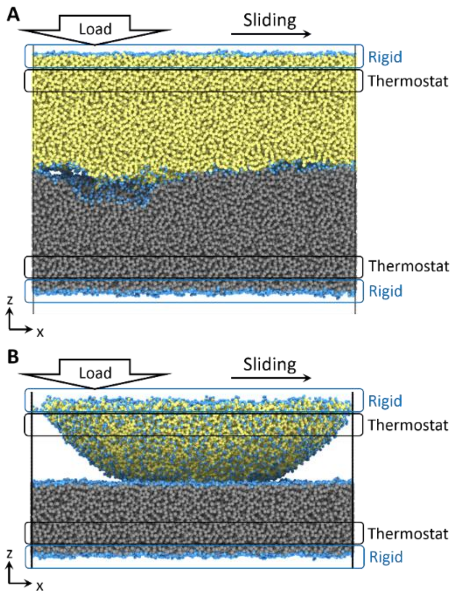

Fig. S1 Simulation model. (**A**) rough-surface and (**B**) smooth ball-on-disk contact.

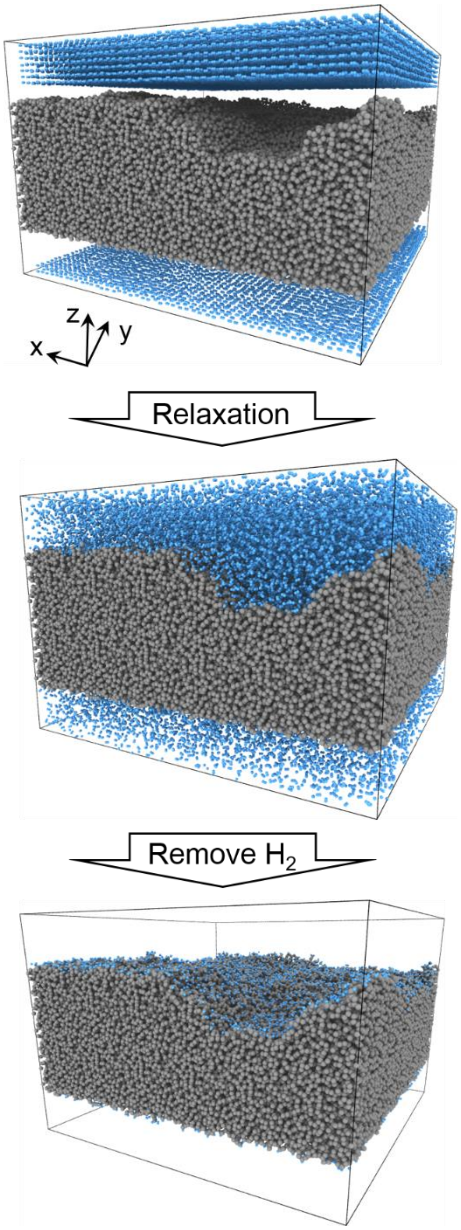

Fig. S2 A typical relaxation process of DLC surface before friction simulation.

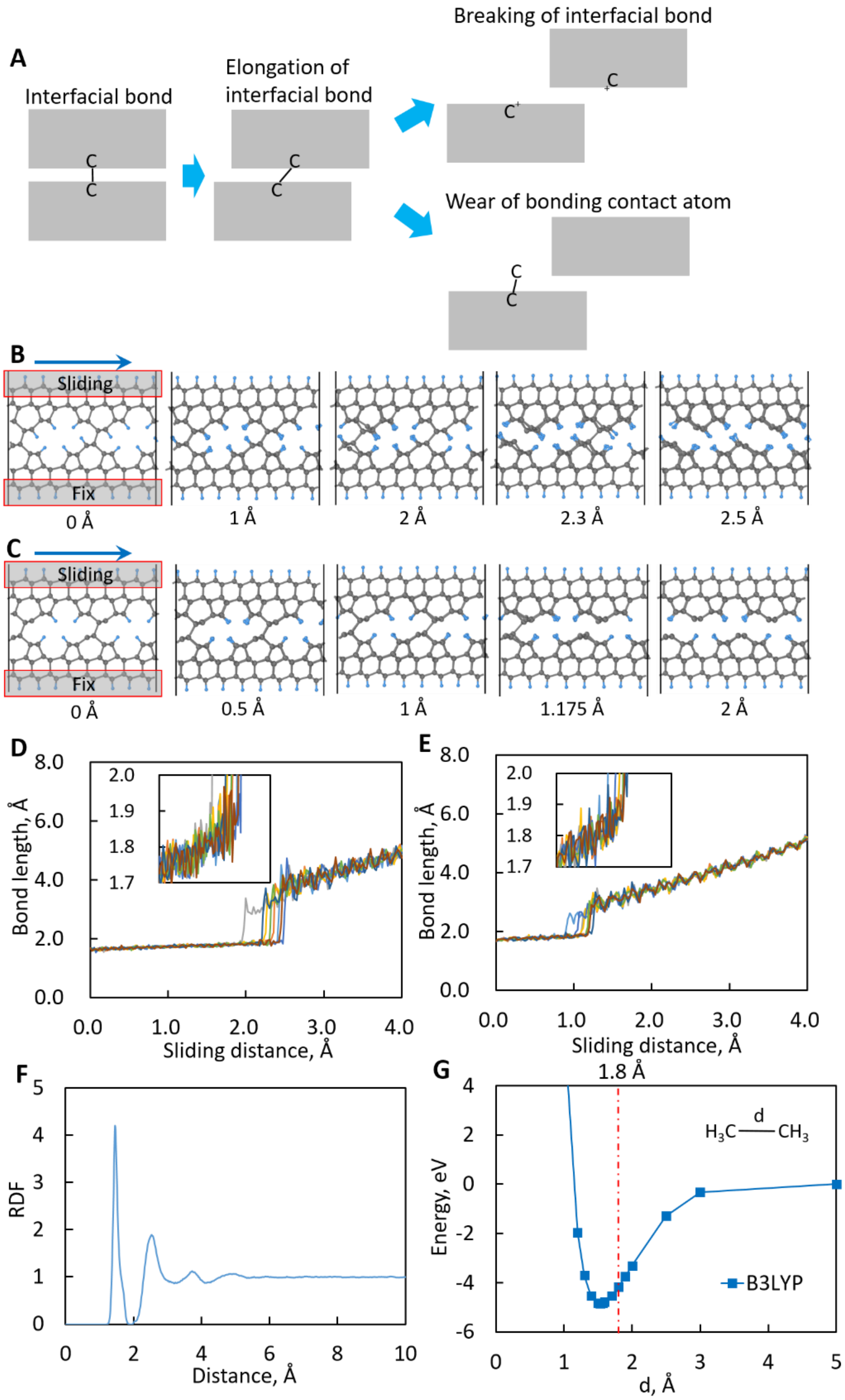

Fig. S3 Estimation of W_τ. (**A**) Schematic illustration of interfacial C-C bond breaking process. (**B**) and (**C**) Extra MD simulations of modified diamond surfaces with totally 8 interfacial C-C bonds at the interface. Surfaces in (**B**) and (**C**) are fully and partly terminated by hydrogen termination, respectively. Bottom

layer of lower diamond substrate is fixed, while top layer of upper diamond substrate is rigid and slid forcibly at a speed of 100 m/s. (**D**) and (**E**) show the instantaneous bond lengths of interfacial C-C bonds with the sliding distance for simulation of (**B**) and (**C**), respectively. The insert in (**D**) and (**E**) clearly show that all of the interfacial C-C bonds break when the bond lengths approach about 1.8-1.9 Å. (**F**) Radial distribution function (RDF) of DLC bulk. (**G**) Potential energy curve of a C-C single bond in ethane molecules obtained by first-principle calculation. Here the density functional theory is used with B3LYP hybrid functional and cc-pVDZ basis set.

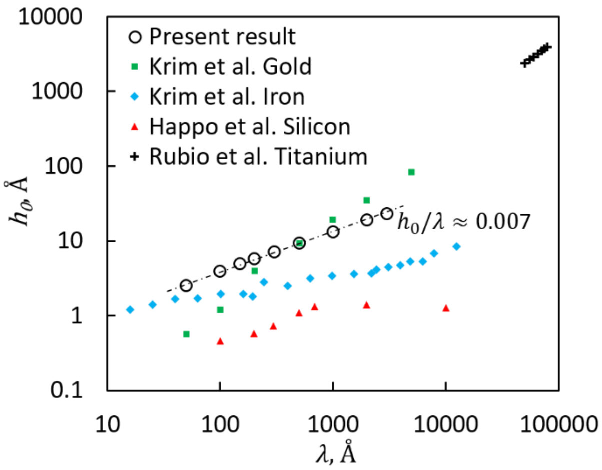

Fig. S4 Scale dependence of rms roughness (h_0). Black open circles are the results for presently created self-affine rough surfaces. The rest results are obtained from experiments by Krim *et al.*[12], Happo *et al.*[13], and Rubio *et al.*[14].

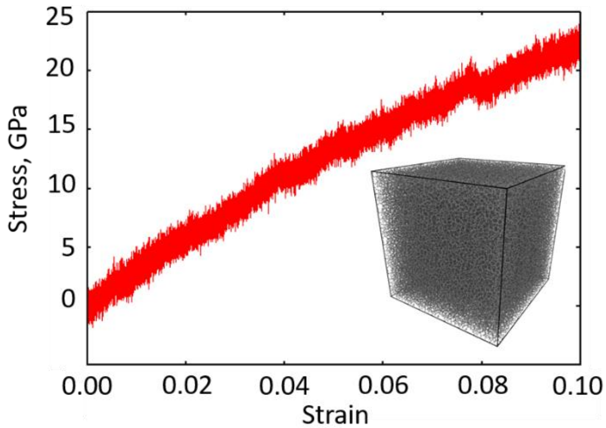

Fig. S5 Stress-strain curve of DLC bulk whose density and hybridizations are the same as the DLC substrate used in friction simulations (ratios of *sp*^{3}-, *sp*^{2}-, and *sp*-hybridized carbon are 39.4%, 60.4%, and 0.2%, respectively, while density is 2.984 g/cm^3).

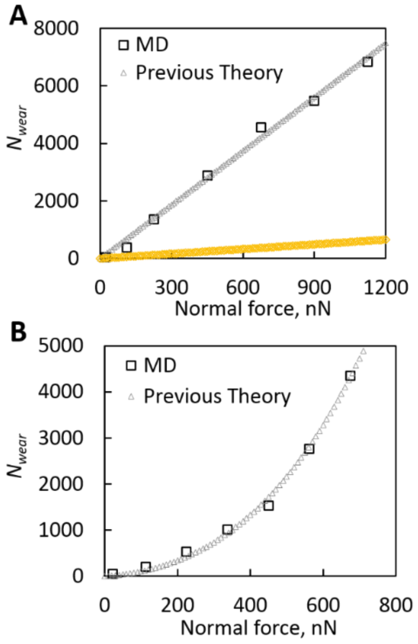

Fig. S6 Comparison of Eq. 1 with MD simulations. Open triangles show the fitting results for (**A**) rough-surface contact by using ΔU_{act} = 0.2965 eV and ΔV_{act} = 1.0001 Å^3 for (**B**) ball-on-disk contact by using and U_{act} = 0.3581 eV and ΔV_{act} = 0.9233 Å^3. Open diamonds in (**A**) shows the prediction results if U_{act} = 0.3581 eV and ΔV_{act} = 0.9233 Å^3 (the extracted values from (**B**)) are used, showing that the previous wear law cannot apply to both contacts with a same set of fitting parameters.

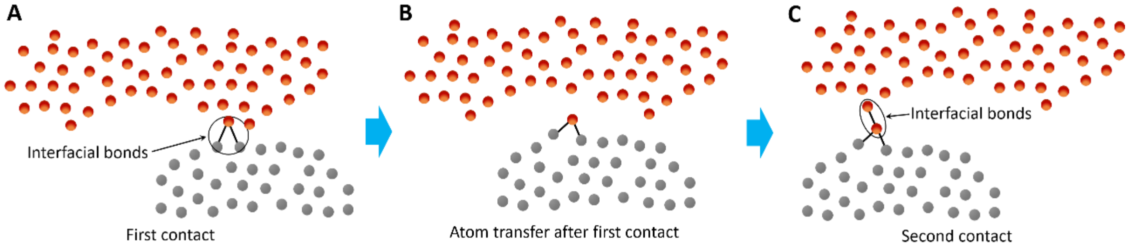

Fig. S7 Recognition of interfacial chemical bond during continuous sliding. (**A**) First contact between two substrates where one atom of the upper substrate has two interfacial chemical bonds with two atoms of the lower substrate. (**B**) The bonding atom of the upper substrate transfers to the lower substrate. (**C**) When two substrates come into contact secondly and interfacial reaction occurs, here only the bond between the post-transferred atom and the upper substrate should be recognized as "interfacial bond" because the post-transferred atom has been belonging to the lower substrate already.

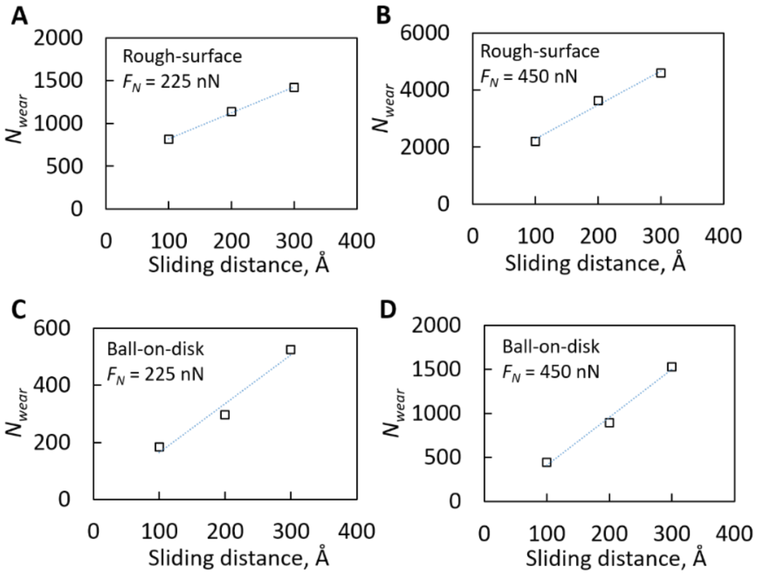

Fig. S8 N_{wear} as a function of sliding distance. (**A**) and (**B**) are the results for rough-surface contact under 225 and 450 nN, and (**C**) and (**D**) are for ball-on-disk contact under 225 and 450 nN, respectively. For the rough-surface contact, the obtained ΔU_{wear} after 100, 200, and 300 Å sliding are 0.910, 0.918, and 0.917 eV, respectively; while for the ball-on-disk contact, the obtained ΔU_{wear} after 100, 200, and 300 Å sliding are 0.913, 0.916, and 0.905 eV, respectively.

Table S1. Parameters of creating self-affine rough surfaces.

	Upper surface	Lower surface
G	0.3	
D	2.5	
γ	1.6	
M	10	
L [Å]	150	
L_s [Å]	2	
k_x	1.9	2.1
k_y	1.9	1.4

References

[1] W. Yan, K. Komvopoulos, *J. Appl. Phys.* **1998**, *84,* 3617.

[2] T. D. B. Jacobs, B. Gotsmann, M. A. Lantz, R. W. Carpick, *Tribol. Lett.* **2010**, *39*, 257.

[3] B. N. J. Persson, *J. Chem. Phys.* **2001**, *115*, 3840.

[4] K. L. Johnson, K. Kendall, A. D. Roberts, *Proc. R. Soc. Lond. A*, **1971**, *324*, 301.

[5] K. L. Johnson, *Proc. Inst. Mech. Eng.* **1982**, *196*, 363.

[6] P. Hänggi, P. Talkner, M. Borkovec, *Rev. Mod. Phys.* **1990**, *62*, 251.

[7] V. Vahdat, K. E. Ryan, P. L. Keating, Y. Jiang, S. P. Adiga, J. D. Schall, K. T. Turner, J. A. Harrison, R. W. Carpick, *ACS Nano* **2014**, *8*, 7027.

[8] Y. Shao, T. D. B. Jacobs, Y. Jiang, K. T. Turner, R. W. Carpick, M. L. Falk, *ACS Appl. Mater. Interfaces* **2017**, *9,* 35333.

[9] J. Liu, Y. Jiang, D. S. Grierson, K. Sridharan, Y. Shao, T. D. B. Jacobs, M. L. Falk, R. W. Carpick, K. T. Turner, *ACS Appl. Mater. Interfaces* **2017**, *9*, 35341.

[10] T. D. B. Jacobs, R. W. Carpick, *Nat. Nanotechnol.* **2013**, *8*, 108.

[11] H. Bhaskaran, B. Gotsmann, A. Sebastian, U. Drechsler, M. A. Lantz, M. Despont, P. Jaroenapibal, R. W. Carpick, Y. Chen, K. Sridharan, *Nat. Nanotechnol.* **2010**, *5*, 181.

[12] J. Krim, I. Heyvaert, C. Van Haesendonck, Y. Bruynseraede, *Phys. Rev. Lett.* **1993**, *70*, 57.

[13] N. Happo, M. Fujiwara, M. Iwamatsu, K. Horii, *Jpn. J. Appl. Phys.* **1998**, *37*, 3951.

[14] M. A. Rubio, C. A. Edwards, A. Dougherty, J. P. Gollub, *Phys. Rev. Lett.* **1989**, *63*, 1685.
